# Adherence to antiretroviral and cancer chemotherapy, and associated factors among patients with HIV–cancer co-morbidity at the Uganda Cancer Institute: a cross sectional study

**DOI:** 10.1186/s12889-023-16387-z

**Published:** 2023-07-28

**Authors:** Caroline Achieng, Nelson Bunani, Joseph Kagaayi, Fred Nuwaha

**Affiliations:** 1 Makerere University School of Public Health, Kampala, Uganda; 2 Uganda Heart Institute, Kampala, Uganda; 3 Rakai Health Sciences Program, Kalisizo, Uganda

**Keywords:** HIV, Cancer, Comorbidity, Uganda, Chemotherapy

## Abstract

**Background:**

Human Immunodeficiency Virus is a major global public health issue affecting millions of people, and sub-Saharan Africa where Uganda lies is disproportionately affected. There has been an increase in cancer among HIV patients which has resulted into use of co-medications that sometimes affect ART and cancer chemotherapy adherence. We aimed to determine adherence to antiretroviral and cancer chemotherapy and the associated factors among patients with HIV-cancer co-morbidity at the Uganda Cancer Institute.

**Methods:**

We conducted a cross-sectional study among 200 randomly selected adult cancer patients infected with HIV and attending the Uganda cancer institute. Antiretroviral and anti-cancer chemotherapy adherence with associated factors were assessed quantitatively. We collected the data using interviewer administered semi-structured questionnaires. Modified Poisson regression with robust standard errors was used to estimate the prevalence ratios (PR) and its 95% confidence intervals (CI) for the factors associated with adherence to Antiretroviral Therapy (ART) and cancer chemotherapy.

**Results:**

Overall, 54% of the study participants adhered to both ART and chemotherapy, and 55% adhered to ART while 65% adhered to cancer chemotherapy. The mean age of the respondents was 42 (SD ± 11years), and a majority, 61% were males.More than half, 56.5% were married and at least 45% had attained a primary level of education. Patients with good adherence to antiretroviral therapy and chemotherapy were 54%. No knowledge of cancer stage (PR = 0.4, 95% CI = 0.3–0.6, P < 0.0001), having an AIDS defining cancer (PR = 0.7, 95% CI = 0.5–0.9, P = 0.005), ART clinic in district not near Uganda Cancer Institute (PR = 0.7,95% CI = 0.8-1.0, P = 0.027) and affordability of cancer chemotherapy (PR = 1.4, 95% CI = 1.0-1.9, P = 0.037) were associated with adherence to both ART and cancer chemotherapy.

**Conclusion:**

Adherence to both ART and cancer chemotherapy was low. Factors significantly associated with adherence were: knowledge of the cancer stage by the patient, the type of cancer diagnosis, source of ART and affordability/ availability of medications. There is a need to provide information on the stage of cancer and adherence counseling to patients. Furthermore, Integration of HIV- cancer care will be necessary for efficient and effective care for the patients.

## Background

Globally, an estimated 37.7 million people were living with HIV in 2020, and about 680,000 died due to AIDS-related illnesses in the same year [1]. Recently, there has been a rise in cancer among the people living with HIV (PLWHIV) with nearly 25–35% of the global HIV-associated deaths occurring among cancer patients and Sub-Saharan Africa (SSA) bears the greatest burden [2, 3]. People Living with HIV (PLWH) are at an elevated risk of developing cancer [4], and have a higher incidence of both AIDS-defining cancers (ADCs) and non-AIDS-defining cancers (NADCs) due to HIV-induced immunodeficiency compared with the general population [5]. Some of the cancers that have been associated with HIV infection include; Kaposi sarcoma, cervical cancer, lymphoma,anal cancer, lung cancer and hepatocellular carcinoma [6].

Uganda had an estimated 1.4 million people living with HIV in 2019, and in the same year approximately 23,000 people died of AIDS-related illnesses [7]. The number of documented new cancer cases has increased over the past years in Uganda with approximately 4,000 cancer patients in registered in 2016 and 1,500 cancer-related deaths [8]. Previous studies in Uganda have established an association between HIV infection and cancers with the prevalence ranging from 23 to 57% [9, 10].

Previous studies have demonstrated that adherence to ART and chemotherapy among HIV-infected cancer patients reduce morbidity associated with opportunistic infections which improves prognosis among these patients [11]. While evidence has found an association between HIV and cancer [10], adherence to medication among cancer patients is sub-optimal. Documented literature elsewhere indicate that adherence to ART and chemotherapy improves patients prognosis [11]. This adherence to care has been associated with; having comorbid conditions, socio-demographic factors, health system factors such as cost of medication, and therapy-related factors such as; the dosing frequency and side effects [12–15].

There is limited data on the ART and chemo adherence in Uganda. Even the available data on ART and cancer chemotherapy has estimated adherence separately. For instance, the average adherence to ART ranges from 50 to 95% [15, 16] which is lower than the Joint United Nations Programme on HIV/AIDS (UNAIDS) cut off, while adherence to chemotherapy is much lower at 14%[17]. Further still, being on ART and taking cancer chemotherapy increases the pill burden, side effects, toxicity which affect adherence [18]. Data on adherence to ART and cancer chemotherapy as well as the associated factors among HIV-infected cancer patients is limited in Uganda. Yet, high levels of adherence to medications have been found to be predictors of better health-related quality of life among HIV-cancer patients [10, 19]. We aimed to determine adherence to antiretroviral and cancer chemotherapy and the associated factors among patients with HIV-cancer co-morbidity at the Uganda Cancer Institute (UCI). These findings will inform the interventions aimed at improving adherence to care among HIV-cancer patients in Uganda.

## Methods

### Study design and setting

This study utilized a cross sectional design in which we quantitatively collected data from the patients with HIV-cancer co-morbidity at the UCI. UCI is a public, specialized, tertiary cancer research and treatment center in Uganda. It is located along upper Mulago Hill road on Mulago Hill, central division, Kampala. It is located about 5 km North East of the central business district. The UCI provides services on research, training, consultation, prevention and cancer treatment in areas of Pediatrics, Oncology, Gynecology, Radiotherapy, surgery, pharmacy and recently venturing into bone marrow transplants. The facility has an in-patient’s facility with a capacity of 80 beds and receives on average about 200 patients daily. The inpatient services run for 24 h daily and the outpatient delivers services from Monday to Friday. The facility serves approximately 10 million people from Uganda and the neighboring countries of; The democratic Republic of Congo (DRC), Rwanda, Burundi, Tanzania, south Sudan and Kenya. While services are free at UCI, the institute suffers major chemotherapy and medicine stock-outs because of the growing population it serves, and the patients have to buy the chemotherapy or wait up to when the it is freely available. Not all patients who came to UCI could afford treatment, those who could not afford to buy the treatment during the stock-outs missed the chemotherapy and the study sought to investigate such factors.

### Study population

We included a sample of 200 patients who were on both ART and cancer chemotherapy computed using Kish Leslie (1965) formular, and attending the UCI in the months of July, August and September 2018. Uganda Cancer Institute on average receives 6 cancer patients infected with HIV on a daily basis, either in outpatient or inpatients department, this number was approximated to be 30 a week and 120 in a month. Given 3 months of data collection this brought the number to 360 patients. From the finite population, we computed the sample size.These patients were obtained using simple random sampling. Simple random sampling is a method used to select participants randomly from the population in a way that each member of the population has an exactly equal chance of being selected. We first obtained a list of patients with HIV-cancer co-morbidity from the UCI patient database with the guidance of the UCI data manager. This list was used as a sampling frame from which the study participants were then randomly selected using random numbers generated by a computer and interviewed using a pre-coded interviewer administered questionnaire. This questionnaire was formulated with guidelines from a tool used to measure adherence to ART with an African resource-constrained setting in South Africa and the WHO five dimensions of adherence [20]. Informed voluntary consent was sought from the participants and the participants were assured of privacy and confidentiality of the information provided. Participants that agreed to take part in the study were offered consent form to sign and they retained a copy. Any participant who declined to consent were excluded but we continued to sample until we reached the final computed sample of 200 participants. All the participants that consented completed the interview.

### Inclusion and exclusion criteria

We included patients who were at least 18 years of age both male and female, diagnosed with any type of cancer, consented, initiated on chemotherapy and had been on ART for at least six months. We excluded critically ill cancer patients.

### Measures

The outcome variable was adherence to both ART and chemotherapy. This was measured using a self-report recall of missed doses method using the four-item Morisky Medication Adherence Scale that has been used in similar studies in South Africa [21]. The Morisky Medication Adherence Scale measures medication adherence by asking the following four questions: (1) Do you sometimes find it difficult to remember to take your medication? (2) Many patients have troubles in taking their medication doses as prescribed; did you miss any doses in the last 7 days? (3) When you feel better, do you sometimes take a break from your medicine? (4) When you feel worse, do you sometimes stop taking your medicine? Adherence was categorized into adherent or not adherent. A patient was considered to be non-adherent if they responded with a ‘Yes’ to any of the questions. A patient was considered adherent if they said ‘no’ to all the four questions. Independent variables included: age which was collected as a continuous variable; sex which was categorized as male and female, education level categorized as, no education, primary, secondary and tertiary levels; marital status categorized as, single, married, divorced, separated, widowed and not living together; occupation categorized as, none, peasant, self employed and employed; residence categorized as, rural and urban; knowledge of cancer stage categorized as early,late and unknown stages; cancer diagnosis characterized as non-AIDS defining cancers and AIDS defining cancers; last time a patient missed taking pills categorized as ever missed and never missed; ever missed appointment dates categorized as yes and no, source of ARVs categorized as ART clinic nearby UCI, ART clinic near home and others that included ART clinics which are far from the UCI and those who got ART from black market; and chemotherapy affordability categorized into yes and no.

### Data collection and analysis

Data were collected using a tested questionare [20]. Each questionnaire was given a unique numerical identifier, and all completed questionnaires were locked in the principal investigator’s cabinet. The principal investigator (PI) kept the key to the cabinet throughout the study to ensure confidentiality. Data was double entered by trained data entrants. Controls were put in place to rule out any wrong entries or skipped fields. Each questionnaire was thoroughly checked for missing data and errors while still at the data collection area. Data was field edited, coded, cleaned and checked for consistency. Coding was done to clearly identify the required variables for analysis. Data was entered in the EPI-Data version 3.1, transferred to Microsoft excel for cleaning, and then exported to STATA version 15 software for statistical analysis.

### Data analysis

All continuous variables were summarized using means with their standard deviations (SD) while categorical variables were recoded as proportions. Modified Poisson regression with robust variances was used at bivariable and multivariable analysis to identify factors associated with adherence to ART and cancer chemotherapy. Prevalence ratios (PRs) were used to estimate the strength of association between the outcome and indicator variables and associations were tested at a 95% confidence interval (CI). Factors with p-value less than 0.05 at multivariate stage were considered significant.

## Results

### Socio-demographic characteristics of the HIV- Cancer comorbidity patients at the Uganda cancer institute

As depicted in Table 1; of the 200 recruited respondents, 61% (122/200) were males. The mean age of respondents was 42 ± 11years. More so, 43% (86/200) of the respondents were Protestants and 56.5% (113/200) were married. Table 1 further shows that near half, 45% (90/200) had at least attained a primary education and 40% (80/200) were in informal-employment. Additionally, more than half, 55.5% (111/200) were from a rural residence, and the majority 71.0% (142/200) of the participants had AIDS defining cancers. Further at least 64.5% (129/200)of the particpants got ART from a clinic near home.

**Table 1:** Socio-demographic characteristics of HIV-Cancer comorbidity patients at the Uganda Cancer Institute

Variable	N = 200(%)
**Age**	
18–35	66 (33)
36–45	74(37)
46–55	39(19.5)
Over 55 years	21(10.5)
**Sex**	
Female	78(39)
Male	122(61)
**Marital status**	
Single	46(23)
Married	113(56.5)
Divorced	25(12.5)
Others**	16(8)
**Level of education**	
None	28(14)
Primary	90(45)
Secondary	60(30)
Tertiary	22(11)
**Occupation**	
None	48(24)
Peasant	47(23.5)
Self Employed	80(40)
Employed	25(12.5)
**Residence**	
Rural	111(55.5)
Urban	89(44.5)
**Type of Cancer**	
Non-AIDs defining cancers	58 (29.0)
AIDs defining cancer	142 (71.0)
**Knowledge of cancer Stage**	
Early	70 (35.0)
Late	48 (24.0)
Unknown	82 (41.0)
**Ever missed appointment dates**	
Yes	71 (35.5)
No	129 (64.5)
**Source of ARVs**	
ART clinic nearby UCI	33 (16.5)
ART clinic near home	129 (64.5)
Other	38 (19.0)
**Chemotherapy affordable?**	
No	100 (50.0)
Yes	100 (50.0)

Other marital status included; separated, widowed and not living together

Other sources of ARVS included; ART clinics which are far from the UCI and home and those who got ART from black market

### Adherence to ART and cancer chemotherapy

More than half, 54% (107/200) of the respondents adhered to both ART and chemotherapy. Additionally, 55% (110/200) adhered to ART and 65% (129/200) adhered to chemotherapy. See Table 2.

**Table 2:** Adherence to ART and Cancer Chemotherapy among patients with HIV-Cancer comorbidity at the Uganda Cancer Institute

Outcome	Frequency (n = 200)	Percentage (%)	95% CI	
ART Adherence	110	55	48.0	61.8
CHEMO Adherence	129	65	57.6	70.9
Overall Adherence	107	54	46.5	60.4

### Multivariable analysis of the factors associated with adherence to ART and chemotherapy among HIV cancer patients attending Uganda cancer institute

Knowledge of cancer stage, type of cancer diagnosis, source of ARVs and affordability of cancer chemotherapy were significantly associated with adherence to ART and cancer chemotherapy. Respondents who did not know their cancer stage (APR = 0.4, 95%CI = 0.3–0.6, P < 0.0001) were 0.4 times less likely to adhere to their treatments than those in the early stage; Patients with AIDS defining cancers (APR 0.7, 95%CI = 0.5–0.9, P = 0.005) were 0.7 times less likely to adhere to both ART and cancer chemotherapy than those with NADCs. ART clinic near UCI was associated with better adherence to the medications (APR = 0.7, 95%CI = 0.8-1.0, P-value = 0.027). Cancer chemotherapy affordability was positively significant to adherence (APR 1.4, 95%CI = 0.02–0.9, P-value = 0.04). See Table 3.

**Table 3:** Factors associated with adherence to ART and chemotherapy among HIV cancer patients attending Uganda cancer institute

Variable	Adherence (n = 107)		Non-Adherence (n = 93)		UPR (95% CI)	P-value	APR (95% CI)	P-Value
	n	%	n	%				
**Sex**								
Male	63	58.9	59	63.4	1		1	
Female	44	41.1	34	36.6	1.1 (0.9–1.4)	0.506	0.8 (0.6-1.0)	0.056
**Side effects**								
Yes	78	72.9	75	80.6	1		1	
No	29	27.1	18	19.4	1.2 (0.9–1.6)	0.173	1.1 (0.8–1.4)	0.705
**Knowledge of cancer stage**								
Early	49	45.8	21	22.6	1		1	
Late	36	33.6	12	12.9	1.1 (0.9–1.3)	0.547	0.9 (0.7–1.1)	0.239
Unknown	22	20.6	60	64.5	0.4 (0.3–0.6)	0.000*	0.4 (0.3–0.6)	0.000*
**Cancer diagnosis**								
Non-AIDs defining	40	37.4	18	19.4	1		1	
AIDs defining	67	62.6	75	80.6	0.5 (0.4–0.5)	0.000*	0.7 (0.5–0.9)	0.005*
Last time a patient missed taking								
Ever missed	22	20.6	40	43	1		1	
Never missed	85	79.4	53	57	1.7 (1.2–2.5)	0.003*	0.9–1.5	0.125
**Source of ARVs**								
ART clinic nearby UCI	13	12.1	20	21.5	1		1	
ART clinic near home	59	55.1	70	75.3	1.2 (0.7–1.9)	0.529	0.7 (0.8-1.0)	0.027*
Other	35	32.7	3	3.2	2.3 (1.5–3.6)	0.000*	0.9 (0.6–1.4)	0.759
**Chemotherapy affordable?**								
No	35	32.7	65	69.9	1			
Yes	72	67.3	28	30.1	2.1 (1.5–2.8)	0.000*	1.4 (0.02–0.9)	0.037*

*= P-value < 0.05, UPR = unadjusted prevalence ratio, APR = adjusted prevalence ratio, CI = confidence interval

## Discussion

We found adherence to both ART and cancer chemotherapy at 54%. Adherence to ART alone in HIV-cancer patients was 55% while that of cancer chemotherapy alone was (65%). These findings are lower than those in a related previous study conducted in Uganda [15]. These findings could be different because of the presence of the co-morbidity of HIV and cancer where one ailment could affect the adherence to the other. Comparable findings were reported by Greer, Amayol et al. in their study where they found rates of adherence to cancer chemotherapy varying from as low as 46% [13]. These findings are also in-line with a review study for adherence in chronic illness that stated that achieving adherence rates above 80% is difficult even in resource –rich countries [22].

Our study found that individuals who did not know their stage of cancer were less likely to adhere well to both their ART and cancer chemotherapy. This could be because knowledge of one’s cancer stage contributes to the way they adhere to medications. It is important for the patients to know whether the cancer is in an early or late stage so as to plan on medication in time. These findings are different from those in studies that found late cancer stage [23, 24], associated with poor adherence [25–27], and another study that found patients with metastatic cancer more likely to become over-adherent to oral chemotherapy [26].

Our study further found the type of cancer diagnosis significantly associated with ART and cancer chemotherapy adherence. Individuals with the AIDS defining cancers such as Kaposi’s sarcoma, Non Hodgkins lymphoma were less likely to adhere well to their co- medications. These findings suggest that patients with advanced HIV disease or AIDS may not adhere well to their medications; this could be explained by the high pill burden, and possibly because of the presence of other comorbidities which may hinder proper adherence to medications.

Accessing ART from a clinic near UCI was found to be positively associated with both ART and cancer chemotherapy than picking ART from in district away from UCI. This could be because the patients find it easy to access the both treatments since the ART treatment Centre and cancer treatment Centre are near each other. Our study found that affordability of chemotherapy to be positively associated with ART and chemotherapy adherence. This is explained by the fact that, at Uganda Cancer Institute most of the chemotherapy is given at a free cost and patients only buy the drugs if they are out of stock. These findings are comparable to those in studies that found supplying medication to patients from the health facility improved medication adherence ,and reducing patient out-of-pocket expenses was associated with improved drug adherence [28]. Therapies that are high-value on high-risk patients like those with the HIV-cancer comorbidity may affect adherence and lead to undesired adherence and health outcomes [28].

The study found that the patients with both HIV and cancer on both ART and cancer chemotherapy generally felt weak most of the times, experienced a number of treatment challenges including drug side effects, missing their doses, stigma and financial challenges. They experienced a number of hospital visits and hospital admissions. These findings are similar to those in other studies that found disease correlates, such as the number of co morbidities, cancer stage, and nodal involvement, associated with negative medication adherence [23, 24], also treatment factors, such as higher doses of medication, worse side effects, switching therapy types, and higher utilization of medical care were associated with poor adherence [25, 26].

### Strength and limitations

This study collected primary data from the patients with HIV and cancer, which was considered reliable information from which adherence was measured. The method (Moriscky scale) used to estimate adherence was a reliable and validated tool.

Due to limited time and resources, our study did not assess the level of adherence per specific type of cancer which could have overestimated the outcome. Future studies are necessary to determine the level of adherence to ART and Cancer chemotherapy per specific type of cancer.

Additionally, this study risked self report bias as it could have been very hard for some participants remember their cancer stage.Further, future studies should review patient records to confirm the cancer stage.

## Conclusion and implications

Overall, adherence to both ART and cancer chemotherapy was low. Factors that were found with adherence were; Knowledge of the cancer stage by the patient, the type of cancer diagnosis, source of ART and affordability of the medications. Our findings suggest a need for health promotion and adherence counseling to increase knowledge of cancer stages and hence adherence among patients with cancer and HIV-comorbidity. Furthermore, there is need to integrate free and/ or subsidized HIV treatment services with cancer care to ease access by the patients.

## Data Availability

The data used and/or analyzed during the study are available from the corresponding author upon reasonable request.

## Abbreviations

UCI: Uganda Cancer Institute
IRB: Institutional Review Board
HDREC: Higher Degrees Research and Ethics Committee
UBOS: Uganda Bureau of Statistics
MOH: Ministry of Health
